# Influence of Magnetic Fields on Magneto-Aerotaxis

**DOI:** 10.1371/journal.pone.0101150

**Published:** 2014-07-01

**Authors:** Mathieu Bennet, Aongus McCarthy, Dmitri Fix, Matthew R. Edwards, Felix Repp, Peter Vach, John W. C. Dunlop, Metin Sitti, Gerald S. Buller, Stefan Klumpp, Damien Faivre

**Affiliations:** 1 Department of Biomaterials, Max Planck Institute of Colloids and Interfaces, Science Park Golm, Potsdam, Germany; 2 Institute of Photonics and Quantum Sciences, and Scottish Universities Physics Alliance (SUPA), School of Engineering and Physical Sciences, Heriot-Watt University, Edinburgh, United Kingdom; 3 Department of Mechanical Engineering, Carnegie Mellon University, Pittsburgh, Pennsylvania, United States of America; 4 Department of Theory and Bio-systems, Max Planck Institute of Colloids and Interfaces, Science Park Golm, Potsdam, Germany; Centre National de la Recherche Scientifique, Aix-Marseille Université, France

## Abstract

The response of cells to changes in their physico-chemical micro-environment is essential to their survival. For example, bacterial magnetotaxis uses the Earth's magnetic field together with chemical sensing to help microorganisms move towards favoured habitats. The studies of such complex responses are lacking a method that permits the simultaneous mapping of the chemical environment and the response of the organisms, and the ability to generate a controlled physiological magnetic field. We have thus developed a multi-modal microscopy platform that fulfils these requirements. Using simultaneous fluorescence and high-speed imaging in conjunction with diffusion and aerotactic models, we characterized the magneto- aerotaxis of *Magnetospirillum gryphiswaldense*. We assessed the influence of the magnetic field (orientation; strength) on the formation and the dynamic of a micro-aerotactic band (size, dynamic, position). As previously described by models of magnetotaxis, the application of a magnetic field pointing towards the anoxic zone of an oxygen gradient results in an enhanced aerotaxis even down to Earth's magnetic field strength. We found that neither a ten-fold increase of the field strength nor a tilt of 45° resulted in a significant change of the aerotactic efficiency. However, when the field strength is zeroed or when the field angle is tilted to 90°, the magneto-aerotaxis efficiency is drastically reduced. The classical model of magneto-aerotaxis assumes a response proportional to the cosine of the angle difference between the directions of the oxygen gradient and that of the magnetic field. Our experimental evidence however shows that this behaviour is more complex than assumed in this model, thus opening up new avenues for research.

## Introduction

A large number of microorganisms have the ability to respond to their physiological needs by directed motion in their environment. This ability which relies on a motility apparatus is highly energetically demanding [1], and must therefore bestow a subsequent advantage to motile organisms over non-motile ones. In an inhomogeneous environment, this advantage is provided by taxis, i.e. the ability of micro-organisms to sense their immediate surrounding over time. Taxis is essential in several biological functions such as the response of leukocytes to pathogenic stimuli [2] and the survival of bacterial colonies [3]. Bacterial tactic sensing typically influences the motion of bacteria by triggering a change of swimming characteristics (e.g., speed, directional changes, frequency of change of direction) by changes in the environment. The motion of bacteria biased by variation in chemical composition, light, and oxygen concentration has been coined chemotaxis, phototaxis and aerotaxis, respectively. Bacterial aerotaxis was first observed by Engelmann [4] in 1881 and has been thoroughly characterised by Adler [5]. The experiment used by Adler consists of loading the bacteria in a capillary that is sealed at one end and open at the other end. This generates the formation of an oxygen gradient resulting from oxygen diffusion from one end and its consumption by the bacteria [5]–[7]. Using aerotaxis, micro-organisms move towards the optimal oxygen concentration in the gradient and form a highly populated band referred to as “aerotactic band”. Magnetotaxis was introduced by the discovery of magnetic cells [8]. The so-called magnetotactic bacteria are microorganisms whose migration is confined by the local magnetic fields. This specificity is inferred by an intracellular chain of nano-magnets that imparts a magnetic dipole on the cell [9]. The biological advantage provided by the magnetic assembly onto magnetotactic bacteria as opposed to non-magnetic bacteria is the simplification of their taxis for an appropriate environment from three-dimensions to quasi-one-dimension. In fact, the term ‘magnetotaxis’ is wrongly coined, since the orientation of the cells in external fields results from a passive response [10]. Thus, magnetotaxis should rather be thought of as magnetically assisted aerotaxis [11].

The correlative study of the tactic response of microorganisms in controlled physiological environments requires the non-invasive sensing of the chemical micro-environment and the correlative observation of the micro-organisms' response at both single cell and cell population levels. Furthermore, the study of magnetic cells requires the control of strength and orientation of magnetic fields, with a precision in terms of field strength significantly smaller than that of the Earth's field. To date, these requirements have not been satisfied in magneto-aerotaxis assays. These were performed with a spectroscopic setup that did not allow visual observation of the bacteria cells and population. Furthermore, the magnetic field strengths applied and the control over the environment (chemical, magnetic) was not adequate for physiological studies [12], [13].

The work presented here aims at evaluating the key parameters in magneto- aerotaxis of bacteria in a physiological environment. We have studied the magneto-aerotaxis of *Magnetospirillum gryphiswaldense* (MSR-1) [14], a microaerophilic magnetotactic bacterium that serves as model organism for magnetite biomineralization. To remedy the shortcomings of the current techniques, we have devised a platform dedicated to the study of microorganisms' response to micro-environmental changes. The platform is constructed entirely from non-magnetic materials and hosts triaxial Helmholtz coils that generate magnetic fields with a precision of ±5% of the Earth's magnetic field. Our method allows the simultaneous correlative mapping of the micro-environmental properties and recording of the characteristic response of the bacteria at the single cell and the cell population levels. Physiological characteristics and magneto-aerotactic behaviour of the bacteria are calculated using a modified diffusion model and a revised model for magneto-aerotaxis, respectively.

## Results

### Bacteria organisation in an oxygen gradient

**Figure 1:**
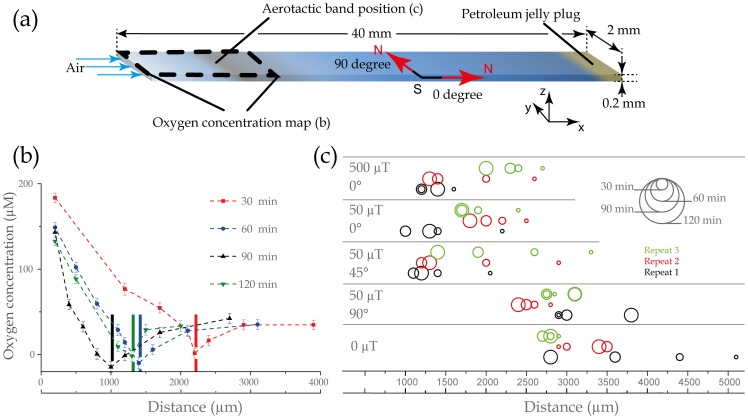
Microcapillary assay and magnetotaxis, experimental results: (a) Schematic of the capillary showing the generation of an oxygen gradient due to air diffusion at one end of the capillary, the consumption by the bacteria at the aerotactic band position shown in (c), and the jelly plug at the other end of the capillary; the area corresponding to the oxygen gradient measured and shown in (b); a schematic of the magnetic field conditions corresponding to 50 µT, at 0° and 90° to the length of the capillary; and the orientation of the x-, y- and z-axis according to how they are referred to in the text. (b) Graph showing the evolution of the oxygen gradient generated and the position of the bacteria in the capillary following the sample preparation (See Figure S7 for other conditions). The measurements are performed after 30 min (red square); 60 min (blue circles); 90 min (black triangles); and 120 min (inversed green triangle). The aerotactic band position is marked by a bar the colour of which corresponds to the colours used to plot the gradients. (c) Position of the aerotactic band when the Earth's magnetic field is cancelled (black squares), a magnetic field of 50 µT is applied and the North pole points towards the anaerobic region (red circles), a magnetic field of 500 µT is applied and the North pole points towards the anaerobic region (blue triangles), a magnetic field of 50 µT is applied and the North pole points at 45° to the capillary long axis towards the anaerobic region (green reverse triangles), a magnetic field of 50 µT is applied and the North pole points at 90° to the capillary long axis (brown squares). Each point represents the position of the aerotactic band at a given time after the beginning of an experiment averaged over three distinct experiments.

MSR-1 bacteria are grown, selected for their motility and transferred to a medium containing an oxygen sensitive dye. They are then loaded into a microcapillary (Figure 1a) that is open at one end (left hand side), through which air diffuses in, and sealed at the other end with petroleum jelly. This results in the formation of an oxygen gradient, and with time an aerotactic band that forms a few millimetres away from the air-water interface. The capillary is placed on the microscope stage under a computer-controlled magnetic field. The aerotactic band position, the distribution of bacteria and the oxygen concentration along the capillary are recorded under five applied magnetic field conditions. *Condition 1*: the Earth's magnetic field is cancelled, which is equivalent to bacteria finding their preferred environment by aerotaxis alone. *Condition 2*: the magnetic field is set to 50 µT, parallel to the oxygen gradient and pointing to the sealed end of the capillary which is equivalent to bacteria placed at the Earth's magnetic pole. *Condition 3*: the magnetic field is set to 500 µT, parallel to the oxygen gradient and pointing to the sealed end of the capillary. *Condition 4*: the magnetic field is set to 50 µT, at 45° to the oxygen gradient and pointing to the sealed end of the capillary which is similar to bacteria placed, for instance, in North Africa, or in Mexico. *Condition 5*: the magnetic field is set to 50 µT, perpendicular to the oxygen gradient (condition 5) which resembles the magnetic field at the equator (the magnetic field strength at the equator is ca. 25 µT).

**Figure 2:**
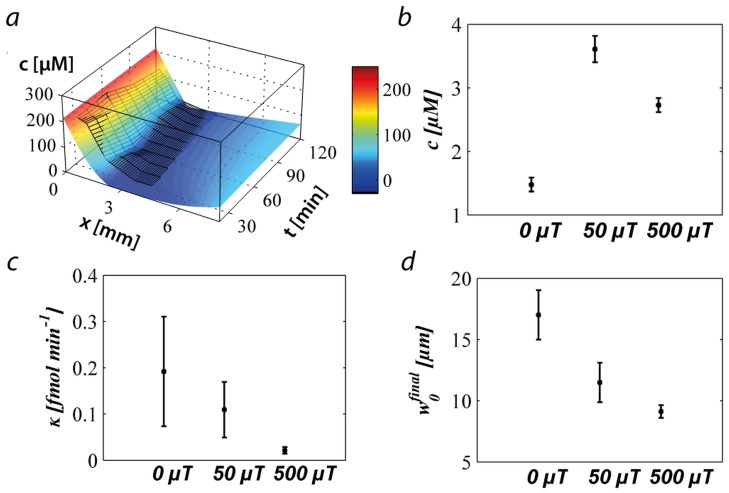
Output of the modified oxygen-diffusion model fitted to the experimental results: (a) Data of the first experiment shown in Figure 1 (black mesh) and the corresponding best fit obtained by solving equation 1 (See also Figure S8); (b) the oxygen concentration average at which the bacteria are localised; (c) the average consumption rate of a bacterium; (d) the final width of the aerotactic band. Panels (b), (c), and (d) are values averaged over three experiments performed on three different days. Each day three experiments were performed, one with no magnetic field, one with a magnetic field of 50 µT parallel to the gradient, and one with a magnetic field of 500 µT parallel to the gradient.

As an example, Figure 2b shows the typical concentration profile of oxygen (O_2_) in the capillary at 30, 60, 90 and 120 minutes after sample preparation performed for condition 2 (See Figure S7 for other conditions). The position of the band over time is indicated by a bar, whose colour matches that of the oxygen measurement. The oxygen concentration at the air-medium interface is 216 µM, then decreases as the position of the aerotactic band is approached and remains relatively constant after the band.

The positions of the aerotactic band over the time course of the experiments and for the three repeats are shown in Figure 1c. Three main trends are observed. If the magnetic field is applied pointing towards the anaerobic region (Figure 1c, 50 µT and 500 µT) or at 45° to the long axis of the capillary (Figure 1c, 50 µT, 45°), the bacteria converge towards a distance of ca. 1.6 mm from the air / solution interface. A convergence also occurs when the magnetic field is cancelled. However this happens much more slowly than in the former cases and after 120 minutes the band is 3 mm away from the open-end of the capillary, i.e. the air-water interface. Finally, we did not observe reproducible aerotactic behaviour when the magnetic field is applied at 90° to the oxygen gradient. In this condition, several bands were observed, some moving towards or away from the oxygen diffusion front, and sometimes bands merged. For this condition, Figure 1c shows the position of the band observed closest to the open-end of the capillary.

We can readily conclude that the spatial organisation of magnetotactic bacteria in a gradient environment is influenced by the presence and the direction of a magnetic field. Whereas the population reaches an equilibrium position after 90 minutes when the field has a component along the gradient, the bacteria do not reach such position within the time frame of the experiment when they are left only with the aerotaxis to guide their quest, experimentally showing the advantage of the combined magneto- aerotaxis in these conditions. Finally, when the magnetic field is perpendicular to the chemical gradient, multiple bands are observed and the system does not converge to an equilibrium position.

### Optimal oxygen concentration and oxygen consumption

As observed in Figure 1b, the ratiometric imaging technique is not suitable for quantitative measurements at the band since the high density of bacteria hinders the fluorescence measurement, resulting in negative values of oxygen concentration. In order to calculate some physiological characteristics about the bacteria, the oxygen concentration profiles corresponding to the first three conditions were fitted using a modified diffusion model (See methods section) [15]. The average oxygen concentration experienced by the cells is 1.5±0.1 µM when there is no magnetic field (condition 1), 2.7±0.1 µM and 3.6±0.2 µM for the bacteria subjected to a magnetic field of 50 µT (condition 2) and 500 µT (condition 3), respectively. These values are in good agreement with previously published preferred oxygen concentrations for microaerophilic bacteria (between 1 and 10 µM) [16]. The oxygen consumption rate, which was the fitted parameter of our model, is ca. 0.1 fmol × min^−1^ per MSR-1 cell and this is comparable to that measured in bulk for *Azospirillum lipoferum* (0.2 fmol × min^−1^ × cell^−1^) [17]. The final width of the aerotactic band is dependent on the magnetic field strength. This is 9.1±0.5 µm at 500 µT, 11±1.6 µm at 50 µT and 17±2 µm at 0 µT.

### Swimming characteristics of bacteria

High-speed videos of the bacteria around the aerotactic band were recorded and analysed (Video S1) for condition 2 to characterise their swimming motility. Table 1 reports the mean velocities measured from high-speed videos of bacteria swimming in the first 100 µm at each side of the band, i.e. in the oxic region and in the anoxic region. The mean velocities in the oxic region and in the anoxic region are significantly different from each other and equal to 22.3±1.1 µm × s^−1^ and 15.7±0.6 µm × s^−1^ respectively. Such difference is probably due to the higher energy level of the cells in the oxic zone (due to the presence of oxygen). The same change of velocity was previously observed for *Desulfovibrio desulfuricans* [18]. Under the magnetic conditions applied (50 µT, 0°), 68% of the bacteria swimming velocities are oriented along the magnetic field lines. In the oxic region, their mean velocities are higher when they return to the band than when they go away from the band (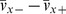 = 2.5±1.5 µm × s^−1^). This difference is not statistically significant in the anoxic region (0.6±1.1 µm × s^−1^). Finally, the mean halting duration before a reversal of the swimming direction is 0.12 s. This value is similar to the one reported by Berg *et al*. [19] for *E. coli* (0.11±0.18 s) and is not significantly different in the oxic and anoxic region of the sample. The change of direction of MSR-1 is similar in the oxic and anoxic regions and equal to ca. 167±9°. This is different from the change of direction of *E. coli* (68±36°) but more similar to that of non-magnetic spirilla, NivaSpi1-NivaSpi5 for which over 50% of the turning angles were measured between 170° and 190° [16]. MSR-1 shares the “run and reverse” motility behaviour typically observed for other spirilla and different from the run and tumble motility of *E. coli* [16], [19].

**Table 1:** Experimental measured mean velocities 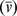, mean of the absolute velocities projected on the long axis of the capillary 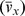, mean of the absolute of the negative velocities (towards the band in the anoxic zone and away from the band in the oxic zone) projected on the long axis of the capillary of the bacteria swimming 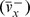, mean of the absolute of the positive velocities (towards the band in the oxic zone and way from the band in the anoxic zone) projected on the long axis of the capillary of the bacteria swimming 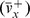, mean halting duration before direction reversal 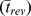, mean change of direction after halting (θ) and associated standard errors (SE) and number of events measured (n).

	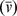 ± SE (n)	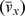 ± SE (n)	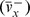 ± SE (n)	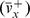 ± SE (n)	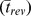 ± SE (n)	θ
	µm/s	µm/s	µm/s	µm/s	s	°
Oxic	22.3±1.1 (78)	14.7±0.9 (78)	13.4±1.4 (66)	10.9±0.7 (60)	0.14±0.03 (21)	166±10° (6)
Anoxic	15.7±0.6 (60)	10.8±0.4 (60)	8.5±0.6 (59)	9.1±0.6 (52)	0.11±0.02 (16)	167±9° (10)

### Model of magneto- aerotaxis

A simple interpretation of our experimental results is that a magnetic field with a significant component along the direction of the oxygen gradient simplifies and speeds up the formation of an aerotactic band by defining a single axis along which the bacteria search for the preferred oxygen concentration. To test this idea more quantitatively, we developed a model for the dynamics of the distribution of bacteria and the oxygen concentration. The model, which is based on earlier models for aerotaxis and magneto-aerotaxis [13], [20], describes movement of the bacteria in one-dimension, distinguishing leftward moving and rightward moving, which swim up or down the oxygen gradient, respectively (See methods section). Sensing of the oxygen gradient is implemented by reversals of the swimming direction with a rate that is increased whenever they swim away from their preferred oxygen concentration, i.e. if the oxygen concentration is too high and the swimming direction is up the gradient or if the oxygen concentration is too low and the swimming direction is down the gradient.

**Figure 3:**
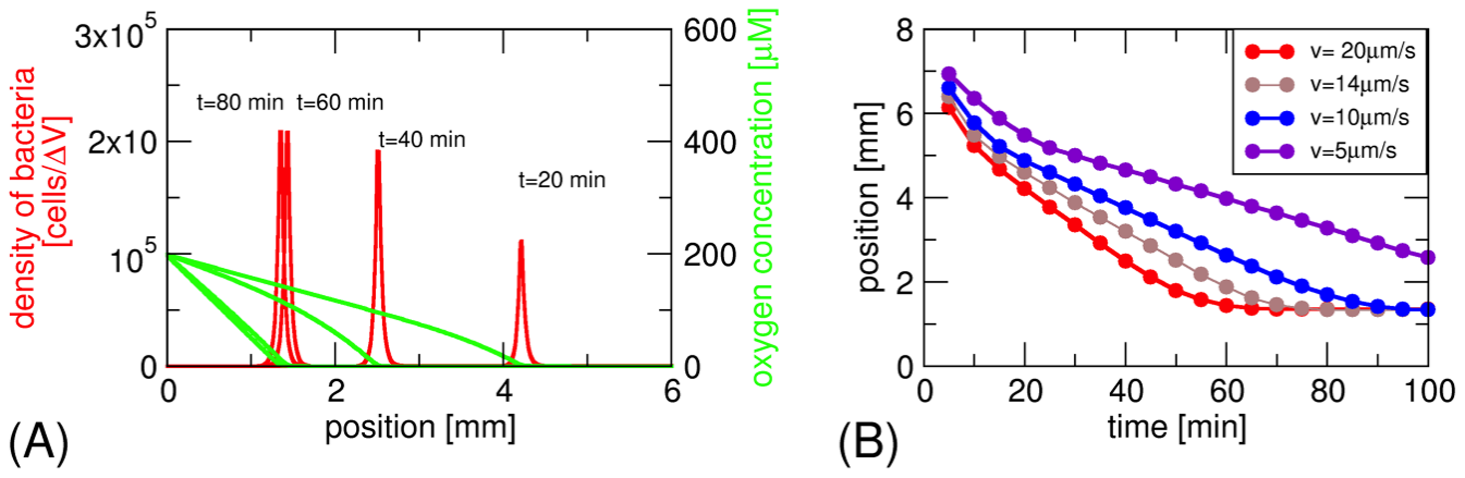
Formation of the aerotactic band in a model for magneto-aerotaxis: (a) Density profile of the bacteria (sharp band, shown in red) and of the oxygen concentration (green) at different time points. The parameter values for the simulations are as given in Table S1, corresponding to a magnetic field parallel to the oxygen gradient. Bacterial densities are given in number of cells per discretization volume ΔV (see Table S1). (b) Position of the aerotactic band as a function of time for different bacterial swimming speeds. A magnetic field at an angle to the oxygen gradient can be interpreted as effectively reducing the swimming speed in the direction of the gradient (the projected swimming speed). Under this simplifying assumption the four speed values correspond to angles of 0°, 45°, 60°, and 75°.

Condition 2 was not included in the magnetotaxis model of Smith *et al.* [13], which does not lead to a stationary band. The numerical solution of the model shows a sharp band of bacteria that forms within 5 min and then moves towards the open end of the capillary within 60 to 80 min (Figures 3a and b). The parameters that we used in these simulations are summarized in Table S1. As in the model of Smith *et al.*, we used a highly simplified description for the switching rates with only two values, a low and a high value. The precise value of these parameters is of lesser importance, as illustrated in Figure S9A, where we show the position of the band from simulations where the switching rates have been increased or decreased (blue data). The ratio of the high and low switching rates has a bigger impact on the dynamics of the band which is slowed down when the ratio is too small. The biggest effect is however seen when the oxygen consumption rate is varied. Changes in the oxygen consumption rate affect both the stationary position and the dynamics of the band (Figure S9B).

Next, we varied the swimming velocity *v* to mimic the effect of a magnetic field under an angle with the gradient (Figure 3b). Reducing the velocity, the shift of the band to its stationary position is slowed, but eventually the stationary position is established. For velocities down to 10 µm × s^−1^, the stationary position is reached within the experimentally observed time scales, but not for 5 µm × s^−1^. Under the strongly simplifying assumption that all bacteria move exactly along the direction given by the magnetic field, the velocity can be related to the angle φ between field direction and gradient direction as 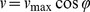. With this assumption, these velocities correspond to angles of 60° and 75°, respectively, thus the behaviour seen in the model is consistent with the experiments where little difference was seen for angles of 0° and 45°. No band will form for an angle of 90°, while the absence of a magnetic field would lead to a mixture of all angles, and thus a slower formation of the band.

**Figure 4:**
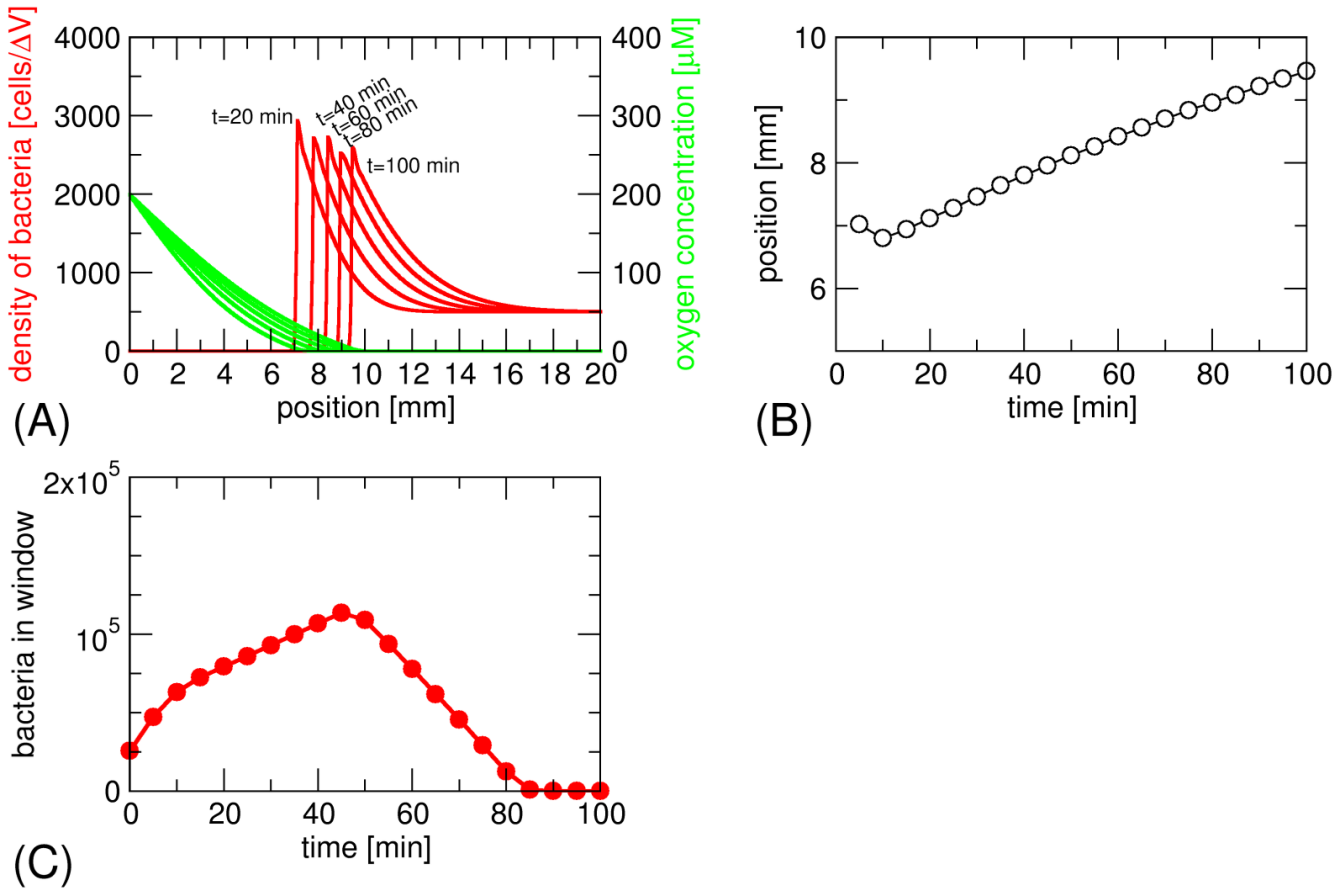
Formation of the aerotactic band in the model for magneto-aerotaxis of Smith *et al*.[13]. (a) The bacterial density shows a high background of cells in the anoxic zone and a region of higher density fleeing the advancing oxygen diffusion front. (b) Position of the bacterial front over time. (c) Simulation of the type of experiment of Smith et al.[13]: number of bacteria in the observation window of a spectroscopic cuvette as the bacterial front travels through it.

As mentioned already, a difference of our model compared to the earlier model of Smith *et al.* is that, in our model, the swimming of bacteria is directed towards increasing oxygen concentration (up the gradient) in the anoxic zone, while in the Smith *et al.* model it is random. We also implemented random swimming in the anoxic zone. In that case, we do not obtain a stationary band. Rather, the bacteria flee from the high oxygen concentration and swim to the closed end of the tube (Figures 4a and 4b). The shape of the band has a characteristic asymmetry and a relatively high constant background density of bacteria is obtained in the anoxic zone. We also note that Smith et al. did not directly observe the density profile, but rather the time dependence of the density seen in an observation window (simulated in Figure 4c), which provides a more convoluted measure of the magneto-aerotactic behaviour.

## Discussion

Using a custom-made microscope platform, we have studied the behaviour of magneto-aerotactic bacteria in a monitored oxygen landscape under physiological magnetic fields. We found that the presence of a magnetic field influences the characteristics of the aerotactic band and its position in an oxygen gradient. In the absence of a magnetic field, the bacteria are only relying on their aerotaxis to find an optimal environment. The population migration is much slower than when the aerotaxis is assisted by a magnetic field. A one-dimensional model of aerotaxis is sufficient to semi-quantitatively describe the behaviour of magnetotactic bacteria in an oxygen gradient; i.e., to show that an increasing average bacteria orientation angle with respect to the oxygen gradient leads to a reduced effective speed towards the favoured habitat, hence a slower organisation. However, current models for magneto-aerotaxis fail to account for the enhancement of the aerotactic response due to the cells being orientated in the environmental gradient.

As shown in Table 1, the effect of the magnetic field on the effective speed is not sufficient to describe the bacteria motion since the speed at which they return to the band, irrespectively of the magnetic field angle, is different in the oxic and anoxic sides of the band. The regions where these measurements have been made (±100 µm from the band) have a relatively similar oxygen concentration with respect to the significant level of oxygen concentration typically used in aerotaxis models [20]. This observation suggests that the speed of the bacteria may in fact depend on the oxygen gradient (ca. - 90 µM × mm^−1^ in the oxic region and ca. +20 µM × mm^−1^ in the anoxic region). With this hypothesis, the broadening of the band width with decreasing magnetic field strength (or increasing angle with respect to the gradient) could be explained by an increasing proportion of bacteria swimming at an angle to the gradient hence reducing their aerotactic capabilities. This behaviour could be due to: the increase of the angle the bacteria swim with respect to the oxygen gradient which is analogous to a decrease in the effective bacteria velocity (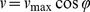); the decrease of their swimming speed due to a shallower apparent gradient (bacteria appear to swim faster in steeper gradients) such as described for *E.coli* by de Gennes [21]; the decrease of the change of oxygen concentration they experience over a given travelled distance.

The current model of magneto-aerotaxis takes into account the first criterion. The development of a comprehensive and quantitative model requires the development of a three-dimensional model that takes into account fluctuations, and dependency of the swimming characteristics and sensing efficiency on the physico-chemical landscape. Such a model will allow the simulation of condition 1 (pure aerotaxis, with no magnetic field) and 5 (hindered aerotaxis).

Whereas the method presented in this study allows for the determination of key physiological characteristics of the magnetotactic bacteria MSR-1, an extension of the method to a larger library of bacteria in general and to magnetotactic bacteria in particular will permit the quantitative comparison of aerotaxis with magnetotaxis and the possible classification of different types of magnetotaxis. Particularly, the comparison between bacteria classified as polar to those classified as axial [11] is of primary importance. This should be done in conjunction with a biochemical study of the chemotactic apparatus in order to develop a fully comprehensive magnetotaxis model.

Finally, magnetotactic bacteria are unique model microorganisms for the study of aerotaxis since their orientation can be non-invasively controlled with an applied magnetic field. The aerotaxis of non-magnetic bacteria is characterised as a biased 3-dimensional random walk. It is therefore technically challenging (e.g.: requires 3-dimensional single cell tracking of numerous events) to gather sufficient data to perform statistically relevant analysis on defined aerotactic scenarios. However, thanks to the magnetic control over the orientation of magnetic micro-organisms, different tactic scenarios can be artificially created (e.g.: different gradient can be tested by orienting the cells) and enough data can be gathered at a low time cost (e.g.: the cells remain at the same focus over the entire field of view) in order to perform statistically relevant analysis.

The proposed method is universal and applicable to any kind of system involving tactic responses. Due to the flexibility of the setup and of the method, the experiments are not limited to the correlative study of magnetotaxis and aerotaxis only. It can also be easily adapted for monitoring the relative importance of other types of taxis such as chemotaxis or phototaxis of magnetic and non-magnetic micro-organisms, and of other categories of responses such as quorum sensing [22], responses to osmotic-stress [23] and adaptation to cyclic changes of the environment [24]. Other key applications for this versatile microscope platform are the study of magnetoreceptor cells under physiological magnetic fields [25] and the study of magnetic microswimmers [26], [27].

## Methods

### Microscope platform

**Figure 5:**
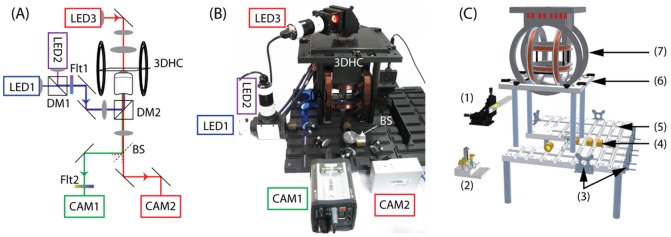
Schematic representation of the optical setup: (a), annotated photograph (b) of the microscope platform showing the two fluorescence excitation sources (LED1 and LED2), the dichroic mirror that combines these two excitation sources (DM1), the excitation filter for fluorescence (Flt1), the dichroic mirror discriminating the fluorescence excitation and the transmission light from the fluorescence emission (DM2), the 3-dimensional Helmholtz coils (3DHC) in which the sample is placed (SMPL) for examination, the microscope objective (OBJ) the transmission light (LED3), the beam splitter (BS), the fluorescence emission filter (Flt2), the fluorescence camera (CAM1) and the high-speed camera (CAM2). Computer aided design (CAD) image (c) of the custom baseplate microscope assembly (Figure S1). The CAD image in (c) shows the microscope baseplate prior to some of the key sub-assemblies being moved into place. Sample stage and attached sample holder (1); sub-baseplate hosting the objective and a dichroic mirror (2); Mounts for attaching of off-plate instruments such as the light sources, cameras, and detectors (3); 35 mm brass barrels used to mount optical elements (4). They are fixed in place using a threaded rod attached to the barrel and passing through the baseplate (Figure S2); Main aluminium baseplate (5); Platform for mounting coils (6); and the 3-dimensional Helmholtz coil set (7).

A schematic of the optical setup and images of the microscope platform are shown Figure 5. The custom-made microscope is constructed around a slotted aluminium baseplate [28] and was designed to provide a non-magnetic platform that can accommodate the magnetic setup without possible disturbance due to magnetic components and the potential to implement multiple light sources and cameras (Note S1 and Figure S1). The fluorescence imaging is implemented in an epifluorescence inverted mode and the sample is illuminated with two optical excitation sources. The triaxial Helmholtz coil set required the use of a flexible and versatile microscope platform in order to provide ease of access and reconfiguration of the optical layouts. The slots act as semi-kinematic alignment channels for optical components housed in, or attached to, custom-designed mechanical holders mounted within 35 mm diameter barrels (Figure S2). In other similar baseplate systems, these optomechanical barrel mounts were held in place on the slots with magnets [28]. Magnets could not be used on the baseplate for this work given the sensitive nature of the measurements being made using computer-controlled magnetic field strengths.

### Optical setup

For the purpose of this study the microscope was equipped with a triaxial Helmholtz coilset and controller (C-SpinCoil-XYZ, Micro Magnetics Inc.), a fluorescence camera (2560×2160 pixels; NeosCMOS, Andor Technology), a high speed camera (540 fps; 1696×1710 pixels; CR3000x2, Optronis), two illumination sources for the fluorescence (pE-100, 400 nm and 470 nm, CoolLED Ltd.) and an illumination source for transmission imaging (pE-100, 635 nm, CoolLED Ltd.) (Note S1). The 3D-axis Helmholtz coils were used to generate DC magnetic fields with a precision of 5% of the Earth's magnetic field (±2.5 µT, manufacturer's specifications). Using this setup, six magnetic conditions were applied under computer control using a LabView based program (LabView, National Instruments). A 3-axis magnetic sensor (Micro Magnetics Inc.) was zeroed in a zero gauss chamber and used to generate a magnetic field that cancels out the ambient magnetic field. The magnetic field was then set to 0 µT, 50 µT or 500 µT along the long axis of the capillary, and to 50 µT at 90° and 45° to the capillary long axis.

### Sample preparation

MSR-1 bacteria cultures were grown in the cultivation medium reported by Heyen and Schüler [29]. A motile population was obtained as follows: 10 ml of a fully grown culture was centrifuged at 4000 rpm for 10 minutes, and suspended in 2 ml of a semisolid medium (0.2% Agar) in a 12 ml plastic culture tube. The tube was then gently filled with 8 ml of semisolid medium in order to avoid convective mixing with the 2 ml of inoculated medium. Due to the bacteria consuming the oxygen at the bottom of the tube and the diffusion of oxygen coming from the ambient atmosphere, an oxygen gradient was formed in the tube and the motile bacteria were able to form a band at a certain level of oxygen. These were collected and inoculated in 10 ml of medium, grown to semi-exponential phase and used to prepare the samples. The bacteria were centrifuged at 4000 rpm for 10 minutes and suspended in the buffer containing 50 µM of the oxygen sensitive dye (Tris(2,2′-bipyridyl)dichlororuthenium(II) hexahydrate, CAS Number 50525-27-4, Sigma-Aldrich) to a final optical density of 0.16 at a wavelength of 565 nm. 0.5 ml of the sample was degassed using nitrogen for 15 minutes and the sample was introduced into a rectangular micro-capillary (#3520–050, Vitrocubes) by capillary forces. One end of the capillary was sealed with petroleum jelly and the capillary was mounted on a microscope slide that was used to hold the sample on the microscope stage.

### Oxygen concentration measurements

The fluorescence of the dye was observed in a fluorospectrometer (Fluoromax 4, Horiba Jobin Yvon). The fluorescence intensity depends on oxygen concentration as described by the Stern-Volmer equation: 
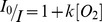
, where *I*_0_ is the fluorescence intensity of the dye in the absence of the quencher (i.e.: oxygen), *I* is the measured fluorescence intensity, *k* is the Stern-Volmer constant and [O_2_] the concentration of oxygen in solution. In order to account for variation in the experimental characteristics (e.g. spatial inhomogeneity of the sample), a ratiometric measurement was performed. This was done by recording fluorescence images of the sample at two different excitation wavelengths. At a wavelength of 470 nm most of the fluorescence detected originates from the dye in the sample. At an excitation wavelength of 400 nm oxygen-insensitive fluorescence from the bacterial medium (Soya peptone, yeast extract) is detected. The detection plane depth is set to the middle of the 200 µm high capillary. For each magnetic condition, the fluorescence images are recorded at different distances from the liquid-air interface in time steps of 30 minutes. A set of data point corresponding to a time step was performed in less than 5 minutes. The experiments corresponding to the 5 magnetic conditions were performed in one day and the total set presented in this work was repeated 3 times. In order to test the validity of the oxygen mapping method, the oxygen concentration in a capillary was calibrated against fluorescence under the conditions used for the magneto-aerotaxis experiment. A peristaltic pump was used to flow some bacterial medium with the fluorescence probe under controlled oxygen conditions (Figure S3). The oxygen content in the medium was adjusted by degassing the solution with nitrogen. The oxygen concentration was read using an oxygen probe (micro TX3 controller and Pst1 microsensor, PreSens-Precision Sensing GmbH). For each fluorescence measurement, four images were recorded: a transmitted-light image; a fluorescence image with excitation at 400 nm; a fluorescence image using an excitation wavelength of 470 nm; and a background image. The camera settings were 1 ms integration time for the transmitted-light image and 150 ms, average of 8 images and 8×8 pixel binning for the fluorescence and background images. The image analyses were performed using a script written in Python [30]. First, an image is created by subtraction of a background image from a fluorescence image. The oxygen concentration at each pixel of the image is calculated using an equation derived from the Stern-Volmer equation which is given by:
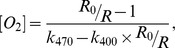
where R_0_ is the intensity ratio I_0,470_/I_0,400_ at [O_2_]  = 0, R is the intensity ratio I_470_/I_400_, k_470_ and k_400_ are the Stern-Volmer constants for the excitation at a wavelength of 400 nm and 470 nm respectively. The calibration curve is shown in Figure S4.

### High-speed videos analysis

The swimming characteristics of the bacteria were determined on each side of the aerotactic band. The band is typically positioned at the very left or right end of the field of view and the videos are recorded. The experiment was performed two hours after the end of the sample preparation (last time point in the aerotaxis experiment) with a magnetic field mimicking the conditions in the Northern hemisphere (Condition 2: 50 µT, North Pole at the sealed end of the capillary). The videos were recorded in the transmission mode at 543 fps. The 2D locations of observed bacteria were determined using an analysis code written in MATLAB (R2012a, Mathworks Inc., Natick, MA, USA), which identified individual bacteria based on an intensity threshold of the contrast of the video frames. Contiguous high and low intensity pixels were grouped into regions whose centroids were identified as bacteria locations. Trajectories were formed by connecting each identified bacteria with the closest bacteria found in a subsequent frame. Trajectories shorter than three bacterial body lengths were discarded, and obvious erroneous trajectories were removed manually. The trajectories were smoothed using a moving average over a ten-frame window. Bacteria direction reversals were identified when a change in direction of more than 90 degrees was observed. The bacteria velocities at particular times were calculated based on the change in position over ten frames. The characteristic average velocity of a population under specific conditions was calculated as the sum of individual bacterium average velocity under these conditions divided by the number of bacteria under these conditions. The mean bacteria swimming speed in the oxic and anoxic region were computed by tracking 78 and 60 bacteria, respectively.

### Model for oxygen diffusion

The evolution of the oxygen concentration *c* with time at different distances from the air/water interface at the end of the capillary can be treated as combination of two processes: (i) diffusion of oxygen in the liquid and (ii) consumption of oxygen by bacteria in the capillary. The free diffusion of a gas into a tube with finite length is well described and results in an analytical solution for oxygen concentration following from Fick's 2^nd^ law [15]. In our system the bacteria represents a sink of oxygen, of which the distribution and position vary over time. Therefore the diffusion equation was modified to 
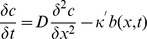
 (Equation 1), where *D* = 2100 µm^2^/s is the diffusion of oxygen in water,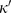 is the concentration dependent consumption rate of the oxygen, and 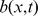 is the number of bacteria in the capillary at the position 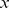 and the time 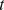 given by:
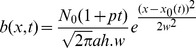



The pre-factor represents the total number of bacteria proliferating at the rate 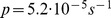. This rate was calculated by measuring the optical density before and after the microscopy experiment. The distribution of the bacteria was modelled as a Gaussian function with average width 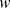 (Figure S5), which changes its centre 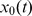
with time. These two parameters were measured by transmission optical microscopy. *N_0_* is the number of bacteria at *t_0_* and is equal to 8.7×10^5^. The consumption 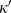 follows the rule by Michaelis-Menten [31]:
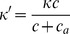
with a constant maximum bacterial activity 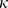. For further calculations the following constants were used: 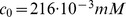 (concentration of oxygen at the air/water interface measured in an equilibrated medium using a calibrated oxygen probe), 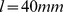 (length of the capillary filled with the sample), 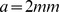 (width of the capillary), 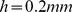 (height of the capillary), and 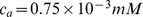 (concentration, at which the activity is half of the maximal oxygen consumption). The above diffusion equation was solved numerically for a set of bacterial activities 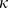 using Mathematica (Wolfram Research, Inc., Mathematica, Version 8.0, Champaign, IL, USA). Due to the optical artefacts hindering the fluorescence measurements around the aerotactic band, the corresponding points were not fitted using the modified oxygen diffusion model. The boundary conditions were set to: 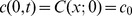 and 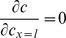. The oxygen concentration of a capillary filled with the measurement medium without bacteria and observed a few minutes after preparation exhibits a shallow oxygen gradient. In order to mimic the experiment, the boundary conditions correspond to a situation where the oxygen concentration at time zero is homogeneous in the capillary and equal to 216 µM, and to a sealed end of the capillary through which we assume no oxygen diffusion. To obtain the best correspondence between the data and theoretical model, a range of bacterial activities was sampled. For each step the adjusted χ^2^ (Equation S1 and Figure S6) is calculated and the maximum value gives the best fitting activity 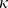.

### Model for magneto- aerotaxis

Here 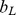 and 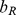 are the numbers of bacteria at position *x* and time *t* that are swimming to the left and right, respectively. Oxygen diffusion and consumption are described by Equation 1. Magneto-aerotaxis was modelled as a one-dimensional process as in previous models [13], [20]. The full model is given by three equations for the local number of bacteria moving to the left (*b_L_*) and moving to the right (*b_R_*) and for the local oxygen concentration (*c*) as:
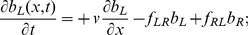


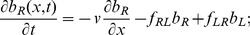


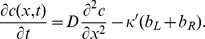



The first two equations describe bacterial swimming (with velocity *v*) and reversals of the swimming direction (with rates *f*_RL_ and *f*_LR_). The last equation describes oxygen diffusion and oxygen consumption by the bacteria and is the same as in the previous section with 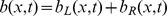. Sensing of the oxygen gradient is taken into account through a dependence of the switching rates on the gradient and the local oxygen concentration. The switching rates can take two values, a basal switching rate and an increased switching rate. The increased value is applied for oxygen concentrations larger than the preferred oxygen concentration *c*_opt_ (*c*>*c*_opt_) if the swimming direction is up the gradient and for *c*<*c*_opt_ if the swimming direction is down the gradient. We note that in this idealised description, bacteria can sense arbitrarily small gradients. (Instead of a preferred oxygen concentration, we have also implemented a concentration range bounded by upper and lower critical concentrations). The parameters used in the simulations are taken directly from the experiments where available and are summarised in Table S1. The model is solved numerically by discretising space and time with units of 20 µm and 0.01 s. The dynamics of the band depends on the initial oxygen profile. To mimic the conditions in the experiments, we start our simulations with an inhomogeneous oxygen profile obtained by letting oxygen diffuse into an empty tube for 20 min and a homogeneous distribution of bacteria with equal probability of swimming to the left or to the right.

## Supporting Information

Figure S1 **Computer aided design (CAD) images of the custom baseplate microscope assembly.** The CAD image in (a) shows the microscope baseplate prior to some of the key sub-assemblies being moved into place. In (b) the sub-baseplate containing the objective, out-of-plane mirror, and dichroic filter, has been moved into place, along with the xyz translation stage that is used for positioning the sample above the objective. The image in (c) is a close-up view from the detector side of the baseplate showing the coils, and sample holder arm, in position.
(TIF)

Figure S2 **Slotted baseplate optomechanical design.** Optical elements such as lenses and spectral filters are usually mounted in cylindrical barrels that are held in place on the slots on the surface of the baseplate, providing ease of alignment and reducing the optic's mechanical degrees of freedom to two: translation along the slot, and roll. The image in (a) shows a magnetic steel barrel mount in position on the slot with a magnet being used to hold it in place. The cross-sectional image of the slotted baseplate in (b) shows the scheme implemented for the microscope platform used in this work– a threaded rod, attached to the 35 mm brass barrel mount and passing through the baseplate, is used to clamp the barrel in place.
(TIF)

Figure S3 **Experimental setup used to calibrate the oxygen concentration as a function of medium fluorescence: solution (1); magnetic stirrer and bar magnet (2); oxygen probe (3); nitrogen supply (4); peristaltic pump (5); camera (6); objective (7); glass capillary (8); light source (9).**
(TIF)

Figure S4
**Calibration curve relating the oxygen concentration to the intensity ratio R0 and R (Black crosses); the dashed line shows the rationale function fitted to the data** 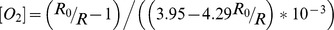
_._
(TIF)

Figure S5 **A transmission image of the aerotactic band (b); the corresponding Gaussian fitted intensity across the image is used to determine the parameters x0 (position of the band) and w (width of the band) of the modified diffusion model.**
(TIF)

Figure S6 **Example of a χ^2^ value versus the oxygen consumption κ.**
(TIF)

Figure S7 **Graph showing the evolution of the oxygen gradient generated and the position of the bacteria in the capillary following the sample preparation.** The measurements are performed after 30 min (red square); 60 min (blue circles); 90 min (black triangles); and 120 min (inversed green triangle).
(TIF)

Figure S8 **Best fit of experimental data obtained using equation 1 (main text) corresponding to experiments performed in condition 1 (a); condition 2 (b); and condition 3 (c).**
(TIF)

Figure S9 **Variation of parameters in the magnetoaerotaxis model: Position of the band with the parameters given in Table S1 (red) and with modified parameters.** (A) Blue data: The high and low switching rates are increased or decreased 3-fold, magenta data: the ratio of the high and low switching rate is increased or decreased 3-fold. (B) Blue: Three-fold increase or decrease of the oxygen consumption rate.
(TIF)

Table S1 **Parameter values used in the numerical calculations.**
(DOCX)

Equation S1
**Degrees of freedom adjusted R-square**. Ny is the number of data points; Np is the number of fitting parameters; y(x) is the data; 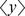 is the mean of the data; and f(x) is the model.(DOCX)

Note S1 **Fabrication of the baseplate and optical components.**
(DOCX)

Video S1 **Tracking of the bacteria on the edge of an aerotactic band.**
(AVI)
