# Supplementary figures and images for: Influence of Magnetic Fields on Magneto-Aerotaxis
Source: PLoS One. 2014 Jul 1;9(7):e101150. doi: 10.1371/journal.pone.0101150 (PMC4077765; doi:10.1371/journal.pone.0101150)

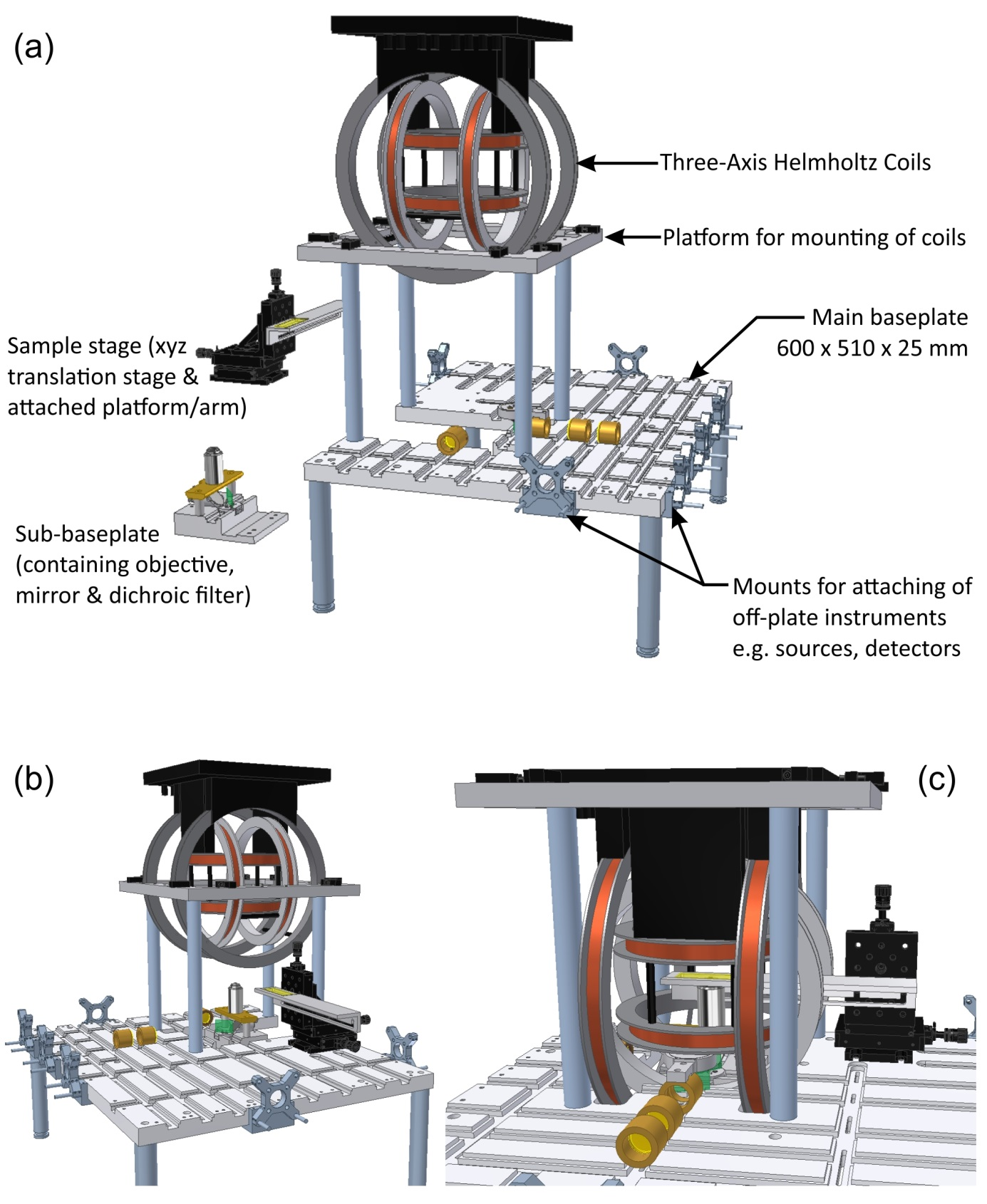

Supplement: Figure S1 — Computer aided design (CAD) images of the custom baseplate microscope assembly. The CAD image in (a) shows the microscope baseplate prior to some of the key sub-assemblies being moved into place. In (b) the sub-baseplate containing the objective, out-of-plane mirror, and dichroic filter, has been moved into place, along with the xyz translation stage that is used for positioning the sample above the objective. The image in (c) is a close-up view from the detector side of the baseplate showing the coils, and sample holder arm, in position. (TIF) [file pone.0101150.s001.tif]

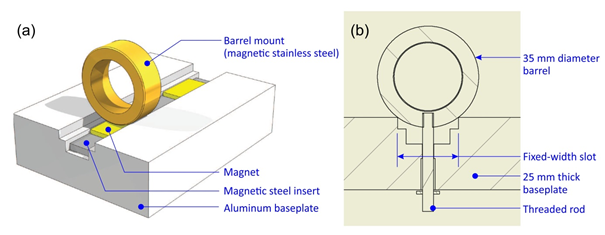

Supplement: Figure S2 — Slotted baseplate optomechanical design. Optical elements such as lenses and spectral filters are usually mounted in cylindrical barrels that are held in place on the slots on the surface of the baseplate, providing ease of alignment and reducing the optic's mechanical degrees of freedom to two: translation along the slot, and roll. The image in (a) shows a magnetic steel barrel mount in position on the slot with a magnet being used to hold it in place. The cross-sectional image of the slotted baseplate in (b) shows the scheme implemented for the microscope platform used in this work– a threaded rod, attached to the 35 mm brass barrel mount and passing through the baseplate, is used to clamp the barrel in place. (TIF) [file pone.0101150.s002.tif]

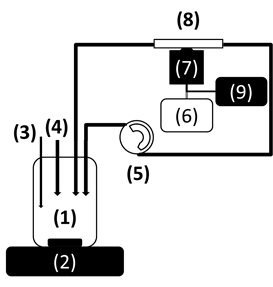

Supplement: Figure S3 — Experimental setup used to calibrate the oxygen concentration as a function of medium fluorescence: solution (1); magnetic stirrer and bar magnet (2); oxygen probe (3); nitrogen supply (4); peristaltic pump (5); camera (6); objective (7); glass capillary (8); light source (9). (TIF) [file pone.0101150.s003.tif]

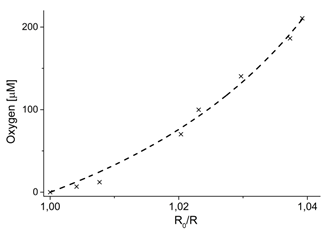

Supplement: Figure S4 — Calibration curve relating the oxygen concentration to the intensity ratio R0 and R (Black crosses); the dashed line shows the rationale function fitted to the data . (TIF) [file pone.0101150.s004.tif]

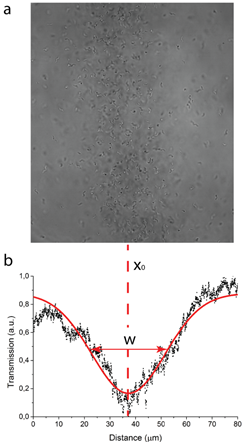

Supplement: Figure S5 — A transmission image of the aerotactic band (b); the corresponding Gaussian fitted intensity across the image is used to determine the parameters x0 (position of the band) and w (width of the band) of the modified diffusion model. (TIF) [file pone.0101150.s005.tif]

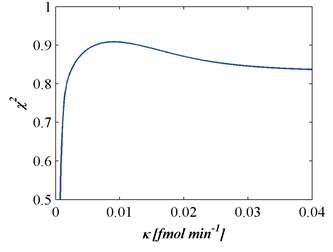

Supplement: Figure S6 — Example of a χ2 value versus the oxygen consumption κ. (TIF) [file pone.0101150.s006.tif]

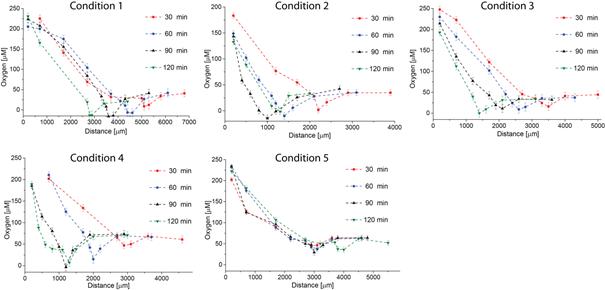

Supplement: Figure S7 — Graph showing the evolution of the oxygen gradient generated and the position of the bacteria in the capillary following the sample preparation. The measurements are performed after 30 min (red square); 60 min (blue circles); 90 min (black triangles); and 120 min (inversed green triangle). (TIF) [file pone.0101150.s007.tif]

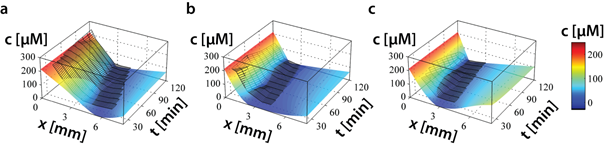

Supplement: Figure S8 — Best fit of experimental data obtained using equation 1 (main text) corresponding to experiments performed in condition 1 (a); condition 2 (b); and condition 3 (c). (TIF) [file pone.0101150.s008.tif]

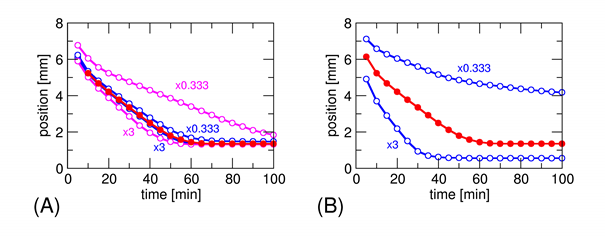

Supplement: Figure S9 — Variation of parameters in the magnetoaerotaxis model: Position of the band with the parameters given in Table S1 (red) and with modified parameters. (A) Blue data: The high and low switching rates are increased or decreased 3-fold, magenta data: the ratio of the high and low switching rate is increased or decreased 3-fold. (B) Blue: Three-fold increase or decrease of the oxygen consumption rate. (TIF) [file pone.0101150.s009.tif]
