# Supplementary material for: Influence of Magnetic Fields on Magneto-Aerotaxis
Source: PLoS One. 2014 Jul 1;9(7):e101150. doi: 10.1371/journal.pone.0101150 (PMC4077765; doi:10.1371/journal.pone.0101150)
Supplement: Table S1 — Parameter values used in the numerical calculations. (DOCX) [file pone.0101150.s010.docx]

Supplementary Table S1

# Influence of Magnetic Fields on Magneto-Aerotaxis Bands

Mathieu Bennet^1^; Aongus McCarthy^2^; Dmitri Fix^1^; Matthew R. Edwards^3^; Felix Repp^1^; Peter Vach^1^; John W. C. Dunlop^1^; Metin Sitti^3^; Gerald S. Buller^2^; Stefan Klumpp^4^; Damien Faivre^1*^

Supplementary Table S1

Table S1: Parameter values used in the numerical calculations.

| **Parameter** | **Value** | **Source** |
| --- | --- | --- |
| Swimming speed *v* | 20 μm × s^-1^ | This study |
| Switching rate (*f*_RL_ and *f*_LR_), basal value | 1/6 s | Smith *et al.*[[1](#_ENREF_1)] |
| Switching rate (*f*_RL_ and *f*_LR_), increased value | 1 s^-1^ | Smith *et al.*[[1](#_ENREF_1)] |
| Oxygen consumption rate κ | 0.01 fmol/min/cell | This study |
| Oxygen concentration at which consumption is half maximal (*c_a_*) | 0.75 μM | See text |
| Oxygen concentration at the open end (*c*_0_) | 200 μM | This study |
| Preferred oxygen concentration (*c*_opt_) | 3 μM | This study |
| Oxygen diffusion coefficient (*D*) | 2100 μm^2^ × s^-1^ | Asfour[[2](#_ENREF_2)] |
| Total number of bacteria | 10^6^ | See text |
| Discretization lengths in numerical solution (Δx) | 20 μm |  |
| Discretization of volume (ΔV) | 8×10^-6^ ml | From Δx and capillary width and height |

1. Smith MJ, Sheehan PE, Perry LL, O'Connor K, Csonka LN, et al. (2006) Quantifying the magnetic advantage in magnetotaxis. Biophysical Journal 91: 1098-1107.

2. Asfour AA (1985) Diffusion: Mass transfer in fluid systems By E. L. Cussler, Cambridge University Press, 1984, 525 pp. AIChE Journal 31: 523-523.
