# Supplementary material for: Influence of Magnetic Fields on Magneto-Aerotaxis
Source: PLoS One. 2014 Jul 1;9(7):e101150. doi: 10.1371/journal.pone.0101150 (PMC4077765; doi:10.1371/journal.pone.0101150)
Supplement: Equation S1 — Degrees of freedom adjusted R-square. Ny is the number of data points; Np is the number of fitting parameters; y(x) is the data; is the mean of the data; and f(x) is the model. (DOCX) [file pone.0101150.s011.docx]

Supplementary Equation S1

# Influence of Magnetic Fields on Magneto-Aerotaxis Bands

Mathieu Bennet^1^; Aongus McCarthy^2^; Dmitri Fix^1^; Matthew R. Edwards^3^; Felix Repp^1^; Peter Vach^1^; John W. C. Dunlop^1^; Metin Sitti^3^; Gerald S. Buller^2^; Stefan Klumpp^4^; Damien Faivre^1*^

Supplementary Equation S1

$$x_{adj.}^{2}=1-\frac{N_{y}-1}{N_{y}-N_{p}-1}*\frac{\sum_{x}\left(y\left(x\right)-f\left(x\right)\right)^{2}}{\sum_{x}\left(y\left(x\right)-\left\langle y\right\rangle\right)^{2}}$$
