# Supplementary material for: Influence of Magnetic Fields on Magneto-Aerotaxis
Source: PLoS One. 2014 Jul 1;9(7):e101150. doi: 10.1371/journal.pone.0101150 (PMC4077765; doi:10.1371/journal.pone.0101150)
Supplement: Note S1 — Fabrication of the baseplate and optical components. (DOCX) [file pone.0101150.s012.docx]

Supplementary information

# Influence of Magnetic Fields on Magneto-Aerotaxis Bands

Mathieu Bennet^1^; Aongus McCarthy^2^; Dmitri Fix^1^; Matthew R. Edwards^3^; Felix Repp^1^; Peter Vach^1^; John W. C. Dunlop^1^; Metin Sitti^3^; Gerald S. Buller^2^; Stefan Klumpp^4^; Damien Faivre^1*^

Supplementary Note S1

*Fabrication of the baseplate*

The optical components and cameras are secured on the microscope baseplate using a combination of custom-made and commercially available optomechanical mounts. The illumination for transmission imaging is held on top of the magnetic setup using a custom-made holder for the LED head. An image of the sample is created on the high-speed camera and the fluorescence camera using an achromatic doublet lens. The wavelengths are spectrally and spatially discriminated using dichroic mirrors and band pass filters. The design and layout of the baseplate was largely driven by the configuration and dimensions of the triaxial Helmholtz coil set. The optimum orientation of the coils, and the mounting of the coils above the main baseplate, was determined by a number of factors including:

- The coils were mounted “upside down”, above the microscope baseplate, as this orientation facilitated the removal of the coils with the minimum disturbance to the optics in the vicinity of the coils. This orientation also enabled them to be lifted up and away from the baseplate.
- The orientation of the coil set about its vertical axis was arranged so as to minimise the intrusion of the coils (with diameters of 200, 250, and 300 mm) into the surface of the main baseplate.

When fixed in position on the microscope, access to the centre of the coil set was restricted. In order to facilitate the loading and positioning of samples, a xyz translation stage was mounted on the baseplate, external to the coil arrangement, with an aluminium arm which extended into the centre of the coils. The capability for attaching/mounting and aligning off-plate instruments (cameras, detectors) and light sources (laser, LED) was provided about the perimeter of the baseplate. The microscope platform was constructed from a 600 mm × 510 mm × 25 mm aluminium plate with a bespoke design of straight, fixed-width slots machined into its surface. The aluminium plate was surface finished with a black anodised coating to help minimise stray light reflections and reduce wear. A threaded rod, attached to the brass barrel mount and passing through the baseplate, was used to clamp the barrel in place (Fig.S2). A variety of commercially available optical components were positioned and aligned on the baseplate using these barrel mounts.

*Optical components*

The fluorescence is implemented in an epifluorescence inverted mode. The two excitation sources are combined using a dichroic mirror mount that could accommodate the LED heads (pE-Combiner, CoolLED). The light is spatially and spectrally filtered using apertures and an excitation filter (HC Tripleband Exciter 482/587, AHF analysentechnik AG). It is reflected off a dichroic mirror (HC Triple Line Beam splitter R405/488/594, AHF analysentechnik AG) and an image of the source is created at the back focal plane of the objective (60X, NA1.2, WI, Planapochromat, Nikon Gmbh). The fluorescence is collected using the same objective and imaged onto the fluorescence camera using a 250 mm effective focal length achromatic doublet lens. The camera is controlled using the Andor iQ software (Andor technology).

The illumination arrangement for transmission imaging is mounted above the magnetic coilset using a custom-made holder for the LED head (LED3). An image of the illumination source is created at the entrance aperture of the condenser lens. The light is collected by the objective and imaged using the achromatic doublet lens. A beam splitter is placed between the imaging lens and the cameras: 50 % of the signal is reflected to the fluorescence camera and 50% is transmitted to the high-speed camera. The high-speed camera is controlled with the software provided by the supplier (Timebench, Optronis).
